# 1,25-Dihydroxyvitamin D3 modulates the phenotype and function of Monocyte derived dendritic cells in cattle

**DOI:** 10.1186/s12917-017-1309-8

**Published:** 2017-12-13

**Authors:** Yolanda Corripio-Miyar, Richard J. Mellanby, Katy Morrison, Tom N. McNeilly

**Affiliations:** 1 Moredun Research Institute, Pentlands Science Park, Bush Loan, Midlothian, UK; 2 The Roslin Institute, Royal (Dick) School of Veterinary Studies, The University of Edinburgh, Midlothian, UK

**Keywords:** Monocyte-derived Dendritic cells, 1,25(OH)_2_D_3_ conditioned MoDCs, Cattle

## Abstract

**Background:**

The active form of the vitamin D_3_, 1,25-Dihydroxyvitamin D_3_ (1,25-(OH)_2_D_3_) has been shown to have major effects not only on physiological processes but also on the regulation of the immune system of vertebrates. Dendritic cells are specialised antigen presenting cells which are in charge of the initiation of T-cell dependant immune responses and as such are key regulators of responses towards pathogens. In this study we set out to evaluate the effects of 1,25-(OH)_2_D_3_ on the phenotype of cattle monocyte-derived dendritic cells (MoDCs) and how the conditioning with this vitamin affects the function of these myeloid cells.

**Results:**

MoDCs were generated from CD14^+^ monocytes with bovine IL-4 and GM-CSF with or without 1,25-(OH)_2_D_3_ supplementation for 10 days. Vitamin D conditioned MoDCs showed a reduced expression of co-stimulatory and antigen presenting molecules, as well as a reduced capability of endocytose ovalbumin. Furthermore, the capacity of MoDCs to induce proliferation in an allogeneic mixed leukocyte reaction was abolished when MoDCs were generated in presence of 1,25-(OH)_2_D_3_. LPS induced maturation of 1,25-(OH)_2_D_3_conditioned MoDCs resulted in lower secretion of IL-12 and higher IL-10 than that observed in MoDCs.

**Conclusions:**

The typical immunotolerant phenotype observed in cattle DCs after exposure to 1,25-(OH)_2_D_3_ has a significant effect on the functionality of these immune cells, inhibiting the T-cell stimulatory capacity of MoDCs. This could have profound implications on how the bovine immune system deals with pathogens, particularly in diseases such as tuberculosis or paratuberculosis.

## Background

Dendritic cells (DCs) are professional antigen-presenting cells (APC) which act as a bridge between innate and adaptive immune responses. DCs patrol the periphery of the immune system in an immature state where they use their unique specialised function to capture and process antigens. Once they receive this maturation signal, they migrate to lymphoid organs and, by secreting cytokines and chemokines, expressing co-stimulatory molecules in their surface and presenting antigens to T cells, are able to initiate a cascade of immune responses [1]. However, DCs not only activate primary immune responses against foreign antigens, they also differentiate non-self from self-antigens and by inducing immunological tolerance in T cells, are able to control autoimmune responses which have the potential to damage the host. The depletion of DCs in animal models breaks self-tolerance of CD4^+^ T cells and is associated with the onset of fatal autoimmune diseases [2].

Given that the induction of immunotolerance could be used to prevent undesirable immune responses for individuals with autoimmune diseases or undergoing transplantations, these cells are being investigated for their potential use as therapeutic targets [3–5]. However, studying the function of DCs in different species can be hampered by the difficulties which arise during the isolation and identification of these cells. Not only are there various DC phenotypes depending on the tissue where they are found, but they also express different markers in different species [6–9]. However, monocytes can be differentiated into dendritic cells (MoDCs) by the addition of IL-4 and GM-CSF [10, 11]. As their function and phenotype are typical of immature DCs, MoDCs are a good substitute to freshly isolated DCs and can be used as in vitro models of innate immune responses.

Recent studies have revealed that the active form of the vitamin D_3_, 1,25-Dihydroxyvitamin D_3_ (1,25-(OH)_2_D_3_), can play an important role in calcium and bone homeostasis and the regulation of the immune system [12–18]. The immune modulatory functions of vitamin D include the regulation of genes involved in host immune defense such as antimicrobial peptides [15], the prevention of the onset of autoimmune diseases models [19], or inhibition of the production of inflammatory cytokines in response to inflammatory or infectious stimuli [20]. However, as an immunomodulator, this hormone can also affect the function and development of monocytes and dendritic cells. As professional antigen presenting cells, DCs require the production of IL-12 to drive naïve T cell to a Th1 phenotype and recent studies have shown a reduction in secretion of IL-12 by DCs which have been treated with high doses of 1,25-(OH)_2_D_3_, leading to an overall reduced functionality [16, 21]. Furthermore, 1,25-(OH)_2_D_3_ is able to down-regulate the expression of MHC-II and co-stimulatory molecules in DCs as well as modulate their cytokine production.

The majority of studies investigating the effects of 1,25-(OH)_2_D_3_ on DCs and the immune system have been carried out in humans [5, 16] or mice [22, 23], with only a few studies carried out in veterinary species. In poultry, Vitamin D has been shown to inhibit proliferation and IFN-γ production by lymphocytes [14], while the addition of 1,25-(OH)_2_D_3_ to chicken macrophages up-regulates the production of nitric oxide by 5-fold, consequently improving the phagocytic nature of these cells [24]. In ruminants, the majority of the work investigating the effects of vitamin D has been focused on nutritional aspects [25], although the role of vitamin D in reproductive fitness [26] and infectious diseases such as bovine tuberculosis [27] and mastitis [28] has also been investigated. In *Mycobacterium bovis* infected cattle it has been shown that 1,25-(OH)_2_D_3_ can inhibit *M. bovis*-specific proliferation of CD4+ and γδ T cells [29, 30]. Furthermore, activation of the vitamin D pathway using a monoclonal antibody to the vitamin D receptor has been shown to suppress *M. bovis*-specific proliferation and interferon gamma (IFN-γ) production of peripheral blood mononuclear cells [27]. In this latter study the suppressive effects were associated with a down-regulation of CD80 expression, suggesting that activation of the vitamin D pathway was associated with a deficiency in antigen presenting cell (APC) function. However, in contrast to work in mice and humans there have been no studies specifically addressing the effects of vitamin D on ruminant APC phenotype and functionality.

In this study we investigate the immunomodulatory effects of 1,25-(OH)_2_D_3_ on cattle DCs. We generated Vitamin D conditioned MoDCs by differentiating bovine CD14 monocytes with IL-4 and GM-CSF in the presence of 1,25-(OH)_2_D_3_ (VitD-MoDCs). We then examined the phenotype and functionality of these VitD-MoDCs and compared to that of standard MoDCs.

## Methods

### Animals

Healthy 6-months old Holstein-Friesian and Ayrshire calves were purchased from two Scottish commercial dairy farms and maintained at Moredun Research Institute (MRI), UK. All animals were kept off pasture for the duration of the experiments. All experiments were approved by the Ethics Committee at MRI and were performed to Home Office Guidelines under Project Licence (PPL 60/3854).

### Isolation of bovine PBMC and in vitro generation of Monocyte derived Dendritc cells (MoDCs)

Blood was collected aseptically into 350 ml blood bags containing 45 ml of Citrate phosphate dextrose-adenine 1 (CPDA-1) stabiliser (Sarstedt, Germany). PBMC were isolated as previously described [31] using density gradient centrifugation by layering whole blood diluted with phosphate buffered saline (PBS) onto Ficoll-Paque™ PLUS (GE Healthcare Life Sciences). Buffy coat was collected and washed three times with PBS and re-suspended in MACS Buffer [PBS + 0.5% foetal bovine serum (FBS, from USA supplied by Sigma-Aldrich, UK)]. CD14 monocytes were positively selected by incubation of PBMC with CD14 MicroBeads (clone TÜK4, Miltenyi Biotech, Germany) in cold MACS buffer for 15 min at 4 °C. Cell-microbead complex were washed twice, resuspended in 3 ml of MACS buffer and purified over an LS column as per manufacturer’s instructions. Purified CD14 monocytes were washed to eliminate any residual microbeads and resuspended in tissue culture medium (RPMI-1640 medium) supplemented with 10% FBS and 50 μM 2-mercaptoethanol, 2 mM L-glutamine, 100 U/ml Penicillin and 100 μg/ml Streptomycin (all from Sigma-Aldrich, UK). Enrichment purity was consistently above 95% as assessed by flow cytometry. Monocyte derived dendritic cells (MoDC) were generated in the presence of bovine GM-CSF and IL-4 (Bovine Dendritic Cell Growth kit, Bio-Rad) for 10 days as previously described [13, 31] with some modifications. Briefly, purified cattle monocytes were seeded in 6-well plates at a concentration of 10^6^cells/ml. Cells were cultured in the presence of 50 μl/ml of Bovine DC Growth Kit and 10 nM 1,25-(OH)_2_D_3_for Vitamin D_3_ conditioned MoDCs (VitD-MoDCs), or 50 μl/ml of Bovine DC Growth Kit only for MoDCs. Fresh medium, cytokines and 1,25-(OH)_2_D_3_ where applicable were replenished on day 3 and 6 of culture. Cells were harvested on day 10.

### Phenotype of MoDCs and VitD-MoDCs

Single colour flow cytometric analysis was carried out to phenotype bovine MoDCs and VitD conditioned MoDCs. After 10 days of culture as detailed above, cells were harvested and following a 10 min blocking step with 20% normal goat serum (NGS, Bio-Rad) in PBS, incubated with the following un-conjugated monoclonal antibodies: CD1b (CC14, Bio-Rad), CD80 (IL-A159, Bio-Rad), CD86 (IL-A190, Bio-Rad) and MHCII-DR (CC108, Bio-Rad) at pre-optimised concentrations for 20 min. Cells were then washed twice with FACS buffer (PBS + 5%FBS + 0.05%NaN_3_) and further incubated for 20 min with a secondary anti-mouse IgG mAb conjugated to Alexa-Fluor® 647 (Invitrogen, Life Technologies, US). After two washes, cells were resuspended in the dead cell stain Sytox Blue (Invitrogen, Life Technologies, US) and immediately a minimum of 10,000 events were acquired using a MACSQuant® Analyzer 10 (Miltenyi Biotech, Germany). Post acquisition gating, including dead cell and doublet cell discrimination, and analysis were carried out using FlowJo vX for Windows 7. Phase contrast images of MoDC and VitD-MoDCs were captured using an Axiovert 200 M inverted microscope (Carl Zeiss Ltd., UK).

### Endocytosis by MoDCs and VitD-MoDCs

One of the main roles of DCs is the sampling of their environment for antigens through endocytosis. Consequently, to assess the effect of 1,25-(OH)_2_D_3_ enrichment on endocytosis, we used DQ-Ovalbumin (DQ-OVA, Molecular Probes, Life Technologies, US), a self-quenched conjugate which exhibits bright green fluorescence upon proteolytic degradation yielding a low background signal. Hence, 10^5^ MoDCs or VitD conditioned MoDCs were incubated with 10 μg/ml of DQ-OVA or medium only for 1 h at 37 °C. After incubation, cells were washed 3 times with FACS buffer and resuspended in Sytox Blue (ThermoFisher) prior to flow cytometry analysis as detailed above.

### Mixed leukocyte reaction

In order to define the capability of VitD conditioned MoDCs to stimulate an alloreactive mixed leukocyte reaction (allo-MLR) an experiment was set up using two animals from different breeds which showed high proliferation in preliminary MLRs. PBMC obtained from an Ayrshire calf were used as responder cells, whilst MoDCs generated from a Holstein-Friesian calf were used as the allogeneic stimulators. MoDCs and VitD-MoDCs were generated for 10 days as described earlier and stimulated with or without LPS for 24 h. Cells were harvested and irradiated (60Gy) to ensure that detected proliferation was derived only from PBMCs. Irradiated MoDCs were then resuspended in complete medium with 10% FBS at a concentration of 10^5^cells/ml. Responder PBMC (10^5^ per well) were incubated with irradiated MoDCs/VitDMoDCs at the following ratios, 1:10, 1:20, 1:100, 1:200, 1:400 and 1:1000. Reactions were set up in quadruplicate in U-well microtitre plates in a total volume of 200 μl. Controls consisted of responder PBMC or irradiated stimulator cells in medium alone or with 5 μg/ml of Concanavalin A (ConA, Sigma-Aldrich). Cells were incubated at 37 °C for 4 days after which 50 μl of medium was collected from each replicate/treatment and replenished with fresh complete medium containing methyl-3H thymidine (0.5 μCi per well; Amersham Biosciences UK Ltd., Chalfont St. Giles, Buckinghamshire). Proliferation was measured by the incorporation of methyl-3H thymidine during the final 18 h of culture as previously described [32]. Data are presented as the corrected counts per minute (ccpm) averaged over 3 min.

### Cytokine secretion after LPS stimulation

MoDCs and 1,25(OH)_2_D_3_ conditioned MoDCs were harvested on day 10 and incubated with 1 μg/ml of LPS (0111:B4, Sigma UK) or medium only for 24 h. After stimulation supernatants were collected to analyse cytokine secretion induced by LPS. Capture ELISAs were performed to examine the secretion of IL-1β (Anti-Bovine IL-1β polyclonal capture and detection antibodies, BioRad), IL-10 (clones CC318 and CC320b, BioRad) and IL-12 (clones CC301 and CC326b, BioRad). Standard curves were constructed using recombinant bovine IL-1β (BioRad) and transfected COS-7 cells supernatants for IL-10 [33] or IL-12 [34]. All incubations were carried out at room temperature unless stated and washing steps were performed 6 times with 350 μl washing buffer (PBS + 0.05% Tween 20) using a Thermo Scientific Wellwash™ Versa (ThermoFisher). Briefly, high-binding capacity ELISA plates (Immunolon™ 2HB 96-well microtiter plates, ThermoFisher) plates were coated with capture antibodies at pre-optimised concentrations and incubated over night at 4 °C. Plates were then washed and blocked for 1 h with PBS+ 3% Bovine Serum Albumin (BSA, Sigma). Following a further washing step, 50 μl of supernatants or standards were added in duplicate for 1 h. Plates were then washed and detection antibodies added for 1 h. This was followed by washing and addition of Streptavidin-HRP (Sigma) for 45 min. After the final washing step, 50 μl of SureBlue TMB substrate (Insight Biotechnology, London, UK) was added and the reaction was stopped by the addition of H_2_SO_4_. Absorbance values were read at O.D. 450 nm. All values were blanked corrected and concentrations determined from standard curve.

### Statistical analysis

Statistical analyses were performed using the non-parametric test Kruskal-Wallis and a Dunns multiple comparison post hoc test in the case of not normally distributed data or One-Way ANOVA and a Tukey post hoc test when data were normally distributed. All analyses were carried out within the Minitab version 17 statistical package, with *p* < 0.05 considered significant.

## Results

### 1,25(OH)_2_D_3_ conditioning influences the phenotype and endocytic capabilities of bovine MoDCs

**Fig. 1:**
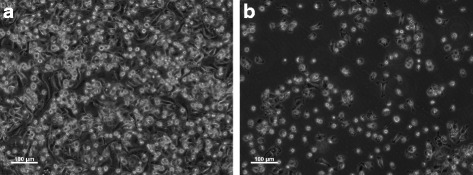
Cattle CD14 + ve monocytes differentiated in the presence of 1,25 (OH)_2_D_3_. MoDCs were generated as detailed in Material and Methods with or without 1,25(OH)_2_D_3_. All treatments were seeded at a 10^6^cells/ml and supplemented with boIL-4, boGM-CSF with or without 10 nM of 1,25(OH)_2_D_3_ on day 1, day 3 and day 6 of culture. Cells were harvested on day 10. Phase contrast images of MoDCs with (**a**) or without (**b**) 1,25 (OH)_2_D_3_ depicts an increased proportion of cells in wells containing cells differentiated in the presence of 1,25(OH)_2_D_3_

**Fig. 2:**
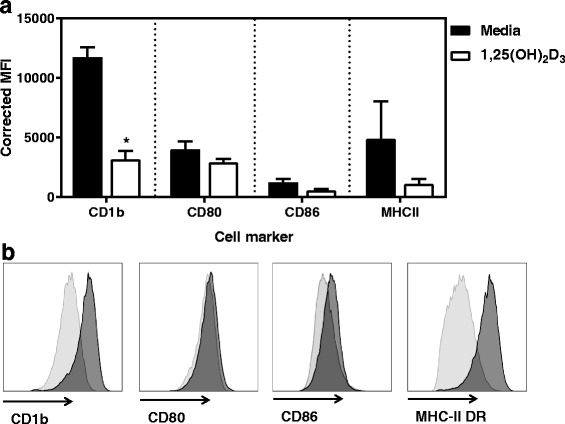
Phenotype of cattle MoDCs differentiated in the presence or absence of 1,25 (OH)_2_D_3_. Phenotype of MoDCs or 1,25 (OH)_2_D_3_ conditioned MoDCS derived from cattle CD14 + ve monocytes was analysed by flow cytometry. MoDCs were single stained with primary mAbs to CD1b, CD80, CD86 or MHC-II DR and then stained with Alexa Fluor 647 IgG secondary antibody. Live, single gated cells were assessed for expression of these markers. **a** Data shown are the average ± the SE of the corrected median fluorescence intensity (MFI) for MoDCs (black bars) and 1,25 (OH)_2_D_3_ conditioned MoDCS (white bars). **b** Histogram for a representative animal showing the level of uptake of expression of chosen markers for MoDCs (black histograms) or 1,25 (OH)_2_D_3_ conditioned MoDCS (grey histograms). *denotes statistical significance for *p* < 0.05

Monocytes were incubated with boIL-4 and boGM-CSF alone or with 1,25-(OH)_2_D_3_ for a period of 10 days. All cultures were set up with the same starting monocyte number, but interestingly, the supplementation of MoDCs with 1,25-(OH)_2_D_3_ during differentiation clearly improved the survival of the MoDCs. Typically, after the 10 day culture period, around 4.6 times more cells were recovered from the 1,25-(OH)_2_D_3_ conditioned cultures than those differentiated with IL-4 + GM-CSF only (cell recovery expressed as percentage of starting population: 6–30% in MoDCs vs 25–100% in VitD-MoDCs; Fig. 1), showing the need for supplementation with 1,25-(OH)_2_D_3_ throughout the differentiation process. In order to determine if 1,25(OH)_2_D_3_ conditioning affected the phenotype and function of MoDCs, we initially investigated the expression of antigen presenting and co-stimulatory molecules which are typically up-regulated in CD14 monocytes cultured with GM-CSF and IL-4 alone. As expected, MoDCs expressed high levels of CD1b, CD80, CD86 and MHC-II (Fig. 2). However, when MoDCs were supplemented with 1,25(OH)_2_D_3_ during differentiation, the expression of all four markers remained consistently lower than in MoDCs. Although this reduced MFI was observed for all four markers, only CD1b was significantly down-regulated in VitD-MoDCs when compared to MoDCs (*p* = 0.016).

**Fig. 3:**
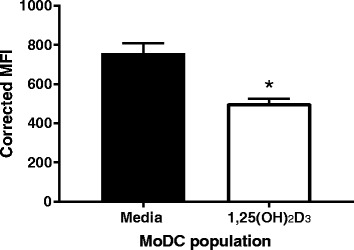
The endocytic capabilities of cattle MoDCs are diminished by conditioning with 1,25(OH)_2_D_3_. MoDCs were differentiated from cattle CD14 monocytes in the absence (black bars) or presence (white bars) of 1,25(OH)_2_D_3_ for 10 days. After harvesting, cells were incubated for 1 h with DQ-OVA and uptake analysed by flow cytometry. Live, single cells were gated and the MFI of each treatment calculated. The results shown are average ± SE of two animals representing the DQ MFI for uptake by MoDCs and 1,25(OH)_2_D_3_ conditioned MoDCs. * denotes statistical significance for *p* < 0.05

Immature DCs have the ability to efficiently uptake antigens by endocytosis, consequently in order to assess the endocytic capacity of the VitD-MoDCs, we investigated the uptake of the model antigen ovalbumin (OVA), a protein taken up by clathrin-coated pits in dendritic cells [35]. 1,25(OH)_2_D_3_ conditioned MoDCs and MoDCs and were incubated with DQ-OVA for 1 h, after which cells were harvested and analysed by flow cytometry. A significantly lower level of internalisation and processing of OVA was observed in VitD-MoDCs when compared MoDCs (Fig. 3, *p* = 0.025).

### Impaired ability of 1,25(OH)_2_D_3_ conditioned MoDC to induce lymphocyte proliferation

**Fig. 4:**
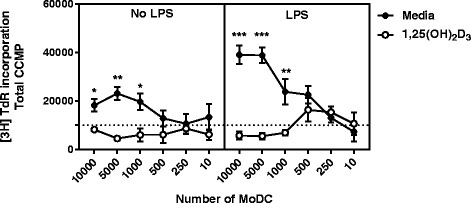
MoDCs differentiated in the presence of 1,25(OH)_2_D_3_ are not able to induce an allogeneic mixed leukocyte reaction. MoDCs differentiated from cattle CD14 monocytes in the presence or absence of 1,25(OH)_2_D_3_ for 10 days. After harvesting, cells incubated with or without LPS for 24 h. Following stimulation, cells were irradiated and cultured in quadruplicate at different ratios with 10^5^responder PBMC for 5 days. Responder PBMC and stimulator MoDCs were incubated in medium only or with ConA (5μg/ml) as controls of proliferation. Proliferation was measured by the incorporation of methyl-3H thymidine ([3H]TdR; 0.5 μCi per well) for the final 18 h of culture. Data are presented as the corrected counts per minute (ccpm) averaged over 3 min. Data shown are the representative of two independent experiments with error bars denoting ± SE. Dashed line denotes the spontaneous proliferation of PBMC with no ConA/stimulator cells. * denotes statistical significance for *p* < 0.05, ** *p* value between 0.001 and 0.01 and *** for *p* < 0.001

We then tested the influence of 1,25(OH)_2_D_3_ conditioning in the ability of MoDCs to induce lymphocyte proliferation in an allo-MLR following LPS maturation. Unstimulated MoDCs were able to significantly increase spontaneous proliferation of PBMC at ratios of 1:10, 1:20 and 1:100 of MoDCs to PBMC (*p* = 0.009, *p* = 0.001, *p* = 0.021 respectively, Fig. 4). In contrast, the incorporation of thymidine by PBMC incubated with unstimulated MoDCs conditioned with 1,25(OH)_2_D_3_ remained below the level of spontaneous proliferation of PBMC only controls (dashed line in Fig. 4), even at high stimulator:responder ratios. This difference was enhanced up to 7-fold when both cell types were matured with LPS prior to setting up the MLR. While LPS stimulated MoDCs were able to generate a high level of proliferation in lymphocytes with as little as 10^3^ MoDCs per 10^5^ PBMC, LPS-stimulated VitD-MoDCs were unable to induce a convincing PBMC proliferative response at any of the MoDC:PBMC ratios tested (LPS VitD conditioned MoDCs vs LPS MoDCS; at 1:10, *p* < 0.001, at 1:20, *p* < 0.001, and at 1:100, *p* = 0.021).

### 1,25(OH)_2_D_3_ enhances LPS driven IL-10 production by MoDCs

**Fig. 5:**
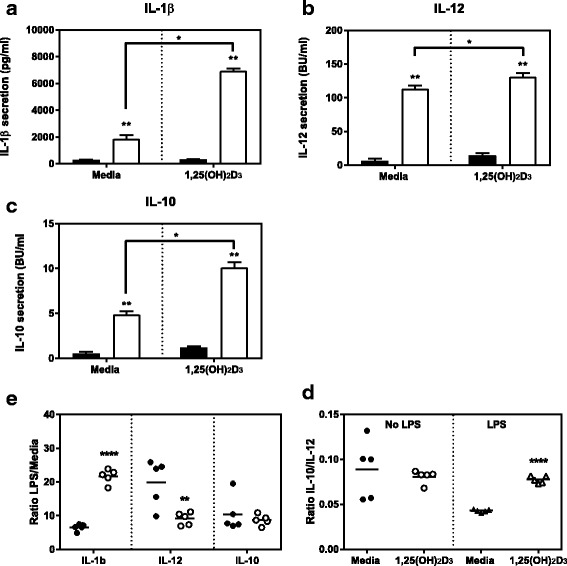
Cytokine secretion by cattle MoDCs differentiated in the presence or absence of 1,25(OH)_2_D_3_. MoDCs differentiated from cattle CD14 + ve monocytes in the presence or absence of 1,25(OH)_2_D_3_ for 10 days. After harvesting, cells incubated with (white bars) or without LPS (black bars) for 24 h. Following stimulation, secretion of IL-1β (**a**), IL-12 (**b**) and IL-10 (**c**) into culture supernatants were measured by ELISA. Data are expressed as the concentration of cytokine in picograms (pg) or biological units (BU) per ml of supernatant. (**d**) Fold increase of cytokine secretion. (**e**) Ratio of IL10/IL12 secretion. Results are shown as the mean values with error bars indicating ± SE from four animals. * denotes statistical significance for *p* < 0.05, ** *p* value between 0.001 and 0.01 and *** for *p* < 0.001

As professional APCs, the main function of DCs is the activation of naive T cells. In order to do so, DCs process antigens and present them on their surface to the T cells. Furthermore, the secretion of cytokines by MoDCs is able to influence the phenotype of the T cells they activate [36]. Consequently, we analysed the LPS induced cytokine secretion by MoDCs differentiated in the presence or absence of 1,25(OH)_2_D_3_ after a stimulation period of 24 h_._ There was no significant difference in the secretion of any of the cytokines between resting MoDCs and VitD-MoDCs (Fig. 5). However, after a 24 h stimulation with LPS, the secretion of IL-1β, IL-10 and IL-12 was significantly upregulated both by MoDCs or VitD-MoDCs (Fig. 5a, b, c; all *p* = 0.009). This LPS driven cytokine secretion was consistently higher in VitD-MoDCs for all three cytokines (all *p* = 0.0122) when compared to MoDCs. When expressed as a fold increase in cytokine release relative to the unstimulated controls, the fold increase in IL-1β secretion by 1,25-(OH)_2_D_3_ conditioned MoDCs was 21.4, while the fold increase for MoDCs was only 6.5 (Fig. 5e, *p*<0.0001). The opposite could be seen for IL-12 secretion, where the MoDCs produced significantly more cytokine than its vitamin D conditioned counterpart (Fig. 5e, *p*=0.01). No significant differences were observed in the fold increase in IL-10 secretion between MoDCs and VitD-MoDCs (Fig. 5e). However, when expressed as a ratio of IL-10/IL-12, MoDCs cells secreted higher levels of IL-12 in comparison to IL-10 after LPS stimulation compared to the 1,25-(OH)_2_D_3_ conditioned MoDCs (Fig. 5d; *p* < 0.0001).

## Discussion

The influence of 1,25-(OH)_2_D_3_ on the function of immune cells has been widely discussed, from their effects on the central nervous system [13], to the modulation of innate immune responses by macrophages [28], or the induction of tolerogenic DCs [16, 18, 22]. However, little information is available on the immunomodulatory effects of 1,25-(OH)_2_D_3_ in ruminants or how they obtain this hormone from the environment.

When exploring the effect of vitamin D_3_ at a cellular level, some studies have focused on its ability to inhibit mitogen or antigen induced secretion of IFNγ in bovine lymphocytes [30, 37], while others have shown that when 1,25-(OH)_2_D_3_ is added to bovine monocyte cultures infected with *Mycobacterium bovis*, NO production is enhanced and apoptosis of antigen-stimulated cells reduced [30]. The production of 1,25-(OH)_2_D_3_ by bovine monocytes has also been reported to modulate iNOS and RANTES expression in LPS stimulated monocytes [38]. However, there is currently little evidence regarding how 1,25-(OH)_2_D_3_ affects other key immune cells such as DCs, which are required to activate naive T cells in order to trigger an effective immune response.

Here we show the profound effects that are caused by 1,25-(OH)_2_D_3_ conditioning during the differentiation of bovine MoDCs. Bovine MoDCs have a distinct phenotype when compared to afferent lymph DCs [35, 39], they express co-stimulatory molecules such as CD1b and MHCII at a higher level than CD14 monocytes all of which are required for antigen presentation. When we investigated the phenotype of MoDCs differentiated in the presence or absence of 1,25-(OH)_2_D_3_, the expression of both markers was lower in the 1,25-(OH)_2_D_3_ conditioned MoDCs, particularly CD1b. This is in agreement with work carried out in other mammalian species, such as human [16, 40] or mice [21], indicating that the presence of 1,25-(OH)_2_D_3_ in the culture medium is able to hinder the complete differentiation of monocytes into MoDCs. Reports on the expression of CD86 and CD80 have been more inconsistent. In some cases CD86 was lowly expressed by VitD-MoDCs and CD80 was unaffected [16]; in other cases, the expression of both cell surface markers is lower when MoDCs are differentiated with 1,25-(OH)_2_D_3_ [23]. During the present study the addition of 1,25-(OH)_2_D_3_ from day 0 impeded the same level of upregulation of CD80 and CD86 seen in MoDCs, a trend seen for all four markers investigated.

The ability to take up antigens is a crucial biological function of dendritic cells. When encountering an antigen, APCs are able to process antigens via the endocytic pathway and present them to quiescent naive T cells, initiating a cascade of immune responses [41]. Consequently, in order to examine if 1,25-(OH)_2_D_3_ conditioned MoDCs are able to endocytose antigens, we investigated the uptake of OVA by clathrin-coated pits [31, 35]. As in human [16, 42], bovine 1,25-(OH)_2_D_3_ conditioned MoDCs are functionally impaired for endocytosis, as a significantly lower level of internalisation of OVA could be observed when compared to cells incubated without vitamin D during the differentiation process.

The key function of DCs, antigen presentation, was not only affected phenotypically by the supplementation of MoDCs with 1,25-(OH)_2_D_3_, but also functionally as seen by the suppression of the T-cell stimulatory capacity in 1,25-(OH)_2_D_3_ conditioned MoDCs. Five days after incubation with allogeneic PBMC, MoDCs were able to induce proliferation with numbers as low as 10^3^ MoDCs per 10^5^ PBMC. However, 1,25-(OH)_2_D_3_ conditioned MoDCs were never able to induce a proliferation higher than background proliferation measured by PBMC incubated in medium only. When maturation was driven by stimulation with LPS for a period of 24 h, this T-cell stimulatory capacity was enhanced in MoDCs while conditioned MoDCs remained lower than the background proliferation. This correlation between phenotype and T-cell stimulatory capacity has been seen in other species [16, 23, 40, 43] and confirms that vitamin D_3_ also fails to activate cattle dendritic cells. Upon TLR4 activation, we also observed a clear up-regulation of IL-12 secretion both in MoDCs and in 1,25-(OH)_2_D_3_ conditioned MoDCs. However, when investigated further, the fold increase in IL-12 with MoDCs was double that seen in 1,25-(OH)_2_D_3_ conditioned MoDCs, a difference also reflected by the low IL-10/IL-12 ratio in MoDCs. Vitamin D3 has been shown to have a negative effect on the production of IL-12 MoDCs after exposure to LPS in human studies [16, 43]. As IL-12 is the main cytokine which drives Th1 differentiation in naive T cells [36], the reduction we observed in IL-12 secretion by 1,25-(OH)_2_D_3_ conditioned MoDCs after LPS stimulation, suggests that these Vitamin D conditioned cells may induce a reduced Th1 phenotype in the T cells they activate.

Secretion of IL-10 by DCs has an important role in immunosuppressive responses and is key to the differentiation of CD4^+^ type 1 T-regulatory (Tr1) cells [44, 45]. Consistent with other murine and human studies [16, 22, 23, 43], we demonstrated that 1,25-(OH)_2_D_3_ conditioned MoDCs secrete relatively higher levels of IL-10 and lower of IL-12 than MoDCs. This indicates a clear suppressing action of vitamin D_3_ on DC development which is able to drive a typical immunotolerant phenotype on cattle DCs. The implications of this on adaptive immune responses in vivo is unclear, although it is known that co-immunization of antigens with supplementary vitamin D results in class-switching of B cells to IgA, suggesting this vitamin can play an important role in modulating bovine adaptive immune responses in vivo [46].

Cattle obtain vitamin D_3_ from either the diet or from photoconversion of 7-dehydrocholesterol in the skin following exposure to UV light from sunlight [47]. As common grassland plants do not contain vitamin D_3_, skin is the principle source of this vitamin in grazing cattle [48]. However, in current agricultural systems a significant proportion of cattle are house under conditions with little or no sunlight and therefore dietary supplementation of vitamin D, usually in the form of vitamin D_3_, is required [25, 49]. Supplementation guidelines are available for cattle which provide daily vitamin D requirements for different classes and ages of cattle [50]. However, these recommendations are largely based on levels of vitamin D required to maintain calcium balance rather than immune function. As vitamin D acts in an endocrine manner for calcium homeostasis, but an intracrine and paracrine manner for many of the non-calcaemic functions of vitamin D [51], it is possible that the requirements of vitamin D for calcium homeostasis and immune function may differ. Consequently current recommendations for vitamin D supplementation in cattle may not be sufficient for optimal immune function. Given the growing body of evidence that vitamin D can modulate immunity in cattle, future research should focus on determining the optimum concentrations of vitamin D_3_ required for immune function, and how variables associated with vitamin D_3_ synthesis in the skin, such as quantity and UV light exposure to the skin, the levels of skin 7-dehydrocholesterol, and skin pigmentation [52], as well as different dietary levels of dietary supplementation of vitamin D, affects the immunity and health status of cattle.

## Conclusion

In summary, the present work demonstrates that conditioning of monocytes with the hormone 1,25-(OH)_2_D_3_ during the monocyte to DC maturation process induces a semi-mature or immunotolerant DC phenotype. As a consequence, the antigen presenting capabilities of these cells is hampered as shown by the reduced ability to endocytose ovalbumin and the inability to induce lymphocyte proliferation in the context of a mixed leukocyte reaction. The effects of Vitamin D_3_-mediated modulation of DC function (both MoDCs and the recently described bovine blood DCs [53]) on pathogen-specific T cell responses should now be investigated, particularly in the context of diseases such as bovine tuberculosis for which a key role for Vitamin D_3_ has been proposed [27].

## Abbreviations

1,25-(OH)2D3: 1,25-Dihydroxyvitamin D3
allo-MLR: Allogeneic Mixed Leukocyte Reaction
ANOVA: Analysis of variance
APC: Antigen presenting cell
BSA: Bovine serum albumin
CPDA-1: Citrate phosphate dextrose-adenine 1
DC: Dendritic cells
FBS: Foetal bovine serum
GM-CSF: Granulocyte macrophages colony-stimulating factor
HRP: Horseradish peroxidase
IFN-γ: Interferon gamma
IgA: Immunoglobulin A
IL-10: Interleukin-10
IL-12: Interleukin-12
IL-1β: Interleukin-1 beta
IL-4: Interleukin 4
iNOS: Inducible nitric oxide synthase
LPS: Lipopolysaccharide
MACS: Magnetic-activated cell sorting
MFI: Median fluorescence intensity
MHC-II: Major histocompatibility complex type II
MoDC: Monocyte derived dendritic cells
NGS: Normal goat serum
OVA: Ovalbumin
PBMC: Peripheral mononuclear cells
PBS: Phosphate buffed saline
RANTES: Regulated on activation, normal t cell expressed and secreted
Th1: T helper 1
Tr1: T-regulatory
UV: Ultraviolet
VitD-MoDCs: Vitamin D conditioned monocyte derived
